# AI-enabled remote learning: promoting educational equity and mental health sustainability in resource-scarce contexts

**DOI:** 10.3389/fpsyg.2026.1705646

**Published:** 2026-04-20

**Authors:** Minghao Mao, Shengguo Zhu, Xinchen Leng, Qixiu Qin

**Affiliations:** 1 College of Foreign Language Education, China West Normal University, Nanchong, China; 2 Law School, Huazhong University of Science and Technology, Wuhan, China; 3 Human Police Academy, Changsha, Hunan, China; 4 College of Social Sciences and Humanities, Northeastern University, Seattle, WA, United States

**Keywords:** academic performance, AI-powered remote learning, educational equity keyword, emotional regulation, mental health intervention

## Abstract

This study examines the impact of artificial intelligence (AI)-powered remote learning platforms on students’ academic performance and mental health in resource-scarce environments, with a focus on educational equity and sustainable mental health outcomes. We conducted a 12-week randomized controlled trial (RCT) to compare the effects of AI-powered platforms and traditional face-to-face teaching on academic performance, emotional regulation, anxiety, depression, and related indicators. The participants were high school and university students, with pre- and post-assessments used to evaluate both academic outcomes and mental health. Data were analyzed using Structural Equation Modeling (SEM) and Bootstrap. The results show that the AI platform significantly improved students’ academic performance (*p* < 0.001), a finding linked to personalized learning pathways and real-time feedback. Moreover, the AI intervention effectively reduced anxiety (*β* = −3.378, *p* < 0.001) and depression (*β* = −2.919, *p* < 0.001). However, its effect on emotional regulation was not statistically meaningful (*p* > 0.05), indicating that while AI systems can alleviate emotional distress, their effect on emotional regulation remains limited. In summary, the study provides evidence that AI-powered learning can narrow equity gaps in resource-scarce contexts, particularly by strengthening academic achievement and reducing negative emotional symptoms. Future studies could extend intervention periods and provide more specialized emotional support to improve both educational and psychological outcomes. These findings highlight the role of educational technology in promoting equity and wellbeing, while also noting the need for context-specific strategies to address emotional regulation.

## 1 Introduction

In the context of the accelerating digitalization of global education, artificial intelligence (AI) has become increasingly integrated into educational systems, reshaping learning processes, teaching practices, and the distribution of educational opportunities. This transformation is particularly pronounced in resource-scarce settings, including rural and economically disadvantaged regions, where persistent shortages of qualified teachers, instructional materials, and mental health support continue to limit the effectiveness of conventional educational approaches. Under such conditions, both educational quality and students’ developmental outcomes are frequently compromised. AI-powered remote learning platforms that provide personalized instruction and real-time feedback have therefore been widely regarded as a promising means of alleviating these structural constraints while promoting educational equity and supporting student wellbeing (Kamalov et al., 2023). Nevertheless, whether such platforms can simultaneously reduce academic disparities and address mental health challenges in resource-scarce contexts remains insufficiently examined through empirical research.

Early attempts to apply AI in education can be traced back to Carbonell’s (1970) development of Intelligent Tutoring Systems (ITSs), which aimed to embed artificial intelligence within computer-assisted instruction. Subsequent studies demonstrated that well-designed ITS could produce learning outcomes comparable to those achieved by human instructors, thereby establishing AI as a legitimate instructional tool rather than a purely supplementary technology (Ma et al., 2014). During this phase, however, evaluations of AI-based educational systems focused predominantly on cognitive outcomes and instructional efficiency, with limited attention to learners’ emotional experiences and psychological wellbeing (Kirkwood and Price, 2013). As digital learning environments have become more widespread, research attention has gradually expanded toward a broader conception of learning that incorporates emotional and motivational dimensions. This emerging perspective reflects a gradual shift from performance-oriented approaches toward more holistic frameworks that integrate cognitive achievement with psychological functioning.

Despite these conceptual developments, the implementation of AI-powered remote learning in resource-scarce educational contexts continues to face significant limitations. One major challenge is the limited availability of localized, adaptable AI platforms, which limits the effectiveness of personalized learning across diverse socio-educational environments. In addition, many online learning systems still inadequately address the emotional dimensions of learning, thereby increasing risks of social isolation, reduced motivation, and adverse mental health outcomes (Atif et al., 2020; Ifenthaler et al., 2021). Existing evidence further suggests that narrowly designed, function-driven platforms may intensify anxiety and cognitive overload, particularly in settings where pedagogical guidance and social support are already insufficient (Ng et al., 2023; Labadze et al., 2023). Against this background, the present study examines the operation of AI-powered remote learning in resource-scarce educational contexts, with a specific focus on its long-term effects on high school and university students’ academic performance and mental health, particularly anxiety, depression, and emotional regulation. By integrating personalized learning pathways, real-time feedback, and emotional regulation support, this study investigates whether AI-enabled platforms can enhance students’ perceptions of educational equity and, in turn, improve both academic and psychological outcomes (Atif et al., 2021; Bhutoria, 2022). To address these questions, a 12-week randomized controlled trial (RCT) was conducted with students from economically disadvantaged regions, using Structural Equation Modeling (SEM) alongside regression and mediation analyses to evaluate the role of AI-powered learning in promoting educational equity and supporting sustainable mental health development. In doing so, the study provides empirical evidence relevant to educational equity (Sustainable Development Goals [SDG] 4) and mental health (SDG 3) within the United Nations Sustainable Development Goals framework (Pedro et al., 2019; Farmanesh et al., 2025).

## 2 Review

### 2.1 Educational inequity in resource-scarce contexts: structural constraints and developmental consequences

Educational inequity in resource-scarce regions constitutes a persistent structural condition shaped by long-standing constraints in infrastructure, technology, and human capital, rather than a temporary or isolated shortage of educational inputs. A growing body of research suggests that students’ limited access to quality education in these contexts reflects deeply embedded institutional and systemic barriers within local education systems. As Sun (2024) observes, although AI technologies are frequently presented as powerful instruments for advancing educational equity, their effectiveness is fundamentally contingent upon the availability of basic technological infrastructure and sustained technical support—conditions that are often lacking in resource-scarce regions. Consequently, AI’s capacity to promote educational equity remains uneven across different socio-educational contexts. This unevenness becomes particularly evident when considering the practical challenges associated with implementing AI-supported educational platforms. Empirical evidence indicates that geographic remoteness and infrastructural fragility substantially constrain the deployment of such technologies. Sharma and Patel (2024) demonstrate that in regions characterized by unstable or limited internet connectivity, AI-based learning systems are difficult to scale effectively, with the unintended effect of reinforcing existing disparities in educational access rather than alleviating them. These infrastructural barriers are further intensified by persistent shortages of qualified teachers who can integrate digital technologies into pedagogical practice. Yi et al. (2024) identify the absence of educators possessing both subject expertise and technological competence as a central driver of educational inequity, particularly in disadvantaged regions where opportunities for professional development are scarce. Against this backdrop, AI-driven personalization is often proposed as a means of compensating for structural disadvantages; however, its realization in resource-scarce settings remains highly conditional. Mah (2024) argues that advanced AI applications based on deep learning and natural language processing depend on stable data infrastructures and continuous technical maintenance, resources that are rarely available in impoverished educational environments. Evidence from adjacent domains reinforces this limitation. Kantipudi et al. (2021), in their analysis of IoT- and cloud-based systems for remote health monitoring, show that such technologies require complex and well-integrated technological ecosystems, suggesting that similar prerequisites apply when these tools are transferred to educational contexts. Without these foundational conditions, AI-enabled personalization is unlikely to move beyond a largely aspirational ideal. Recent discussions on generative AI and sustainable education further highlight the structural nature of educational inequity. Baskara (2024) notes that although generative AI has the potential to support more flexible and innovative learning models in resource-scarce regions, its practical impact is constrained by unresolved infrastructural, institutional, and policy-related challenges. Viewed from this perspective, educational inequity cannot be addressed solely through technological innovation, as it reflects cumulative disadvantages rooted in systemic disparities. Students in resource-scarce regions, therefore, experience developmental constraints that extend beyond immediate learning outcomes, shaping their educational trajectories and future life opportunities. The literature thus points to a critical implication: without confronting the underlying structural conditions of inequality, AI-based educational interventions are unlikely to produce sustainable improvements in educational equity, underscoring the need to examine how these inequities translate into broader developmental risks, particularly for students’ psychological wellbeing.

### 2.2 Educational inequity and adolescent mental health: psychological pathways and cyclical effects

Educational inequity in resource-scarce regions represents a persistent structural condition that extends beyond disparities in academic resources to shape adolescents’ psychological development and wellbeing. Existing empirical research consistently indicates that students in resource-scarce educational environments face elevated risks of anxiety, depression, emotional instability, and reduced self-esteem, reflecting the cumulative effects of limited instructional support, uneven teacher quality, and fragile social support systems (Nashwan et al., 2023). Yi et al. (2024) further demonstrate that educational inequality is frequently accompanied by insufficient access to mental health services, creating a compounded vulnerability in which academic strain and psychological distress reinforce one another. Within such contexts, students are often exposed to prolonged uncertainty regarding academic performance and future prospects, fostering chronic stress and emotional strain. Jacobs et al. (2024) argue that restricted access to quality education contributes to a sense of powerlessness, as students perceive limited control over their educational trajectories, a psychological condition closely associated with heightened anxiety and depressive symptoms.

Beyond individual emotional responses, educational inequity also undermines motivational and social processes essential for healthy adolescent development. Kibibi (2024) notes that the absence of consistent encouragement and emotional support in resource-scarce settings weakens students’ engagement with learning, while repeated academic setbacks contribute to disengagement and emotional exhaustion. Sharma and Patel (2024) similarly highlights that difficulties in social adaptation, combined with recurrent experiences of academic failure, accumulate over time, intensifying psychological distress and reducing students’ capacity to cope with educational demands. These interrelated processes give rise to a reinforcing cycle in which psychological distress constrains learning motivation and academic performance, thereby deepening perceptions of educational exclusion and marginalization. Emphasize that the absence of stable social and emotional support structures is central to the persistence of this cycle, suggesting that mental health outcomes should not be treated as secondary consequences of educational inequality. Instead, psychological wellbeing operates as a key pathway through which structural educational disadvantages translate into broader developmental risks, shaping students’ academic engagement, emotional resilience, and long-term educational trajectories.

### 2.3 AI-powered remote learning as a conditional intervention: potential, constraints, and unresolved gaps

Recent research increasingly positions AI-powered remote learning as a potential intervention to address educational inequity and its associated psychological risks; however, existing evidence suggests that its effectiveness is highly conditional and context-dependent. Studies indicate that AI-enabled platforms can provide personalized learning pathways, adaptive feedback, and flexible access to instructional content, thereby offering partial compensation for the shortage of educational resources in remote and resource-scarce regions. At the same time, the practical deployment of such systems remains constrained by persistent infrastructural limitations, including unreliable internet connectivity, limited device availability, and uneven technological adaptability within local education systems (Tanveer et al., 2020). These constraints significantly restrict the scalability and sustainability of AI-based interventions in resource-scarce contexts.

Research in adjacent domains further highlights both the promise and the limits of AI-enabled support. Chen et al. (2025) and Mohanadas (2025) demonstrate that AI technologies used in remote health monitoring can deliver targeted, individualized support, suggesting analogous potential applications in educational settings, particularly for integrating academic instruction with emotional and psychological assistance. Similarly, Shaik et al. (2022) argue that AI-assisted systems can facilitate personalized mental health interventions, while Higgins and Wilson (2025) emphasize the relevance of AI for strengthening sustainable mental health care frameworks. Evidence from multimodal AI research further suggests that such systems may support the detection of emotional states and early psychological risks, even in remote settings (Igwama et al., 2024; Sabapathi et al., 2025). Despite these advances, several unresolved challenges remain. Zhang (2024) notes that although generative AI technologies offer new possibilities for flexible learning and resource redistribution, their educational impact is constrained by policy fragmentation, institutional capacity, and uneven technological integration, particularly in disadvantaged regions. Singh et al. (2024) similarly emphasize that while AI aligns conceptually with inclusive and Sustainable Development Goals, empirical evidence demonstrating its effectiveness across both educational and psychological domains remains limited. Importantly, existing studies tend to examine educational outcomes and mental health outcomes in isolation, with relatively little attention to how AI-powered remote learning might simultaneously address academic inequity and psychological vulnerability within a unified framework (Khan and Shaikh, 2024). Pachava et al. (2025) further highlight the lack of integrative empirical research that explicitly links AI-enabled educational interventions to both equity-related outcomes and mental health processes in resource-scarce contexts. As a result, despite growing optimism surrounding AI-driven remote learning, the existing literature has not yet reached a clear consensus on whether such interventions can generate sustained improvements in both academic performance and psychological wellbeing, nor on the contextual conditions under which these effects are most likely to emerge. Recent studies highlight the promise of AI-enabled learning environments in diverse settings, including African higher education institutions (Shaban et al., 2025) and global school-based pedagogical initiatives aimed at improving health literacy (Owolabi et al., 2025). Empirical evidence from rural China further suggests that AI devices can enhance teaching quality and learning outcomes, particularly in resource-scarce educational systems (Chen et al., 2025). At the same time, research on AI-driven adaptive learning for sustainable educational transformation emphasizes that the effectiveness of such technologies depends heavily on institutional readiness, pedagogical integration, and long-term system support (Strielkowski et al., 2025). Taken together, these studies indicate that while AI-powered educational technologies hold considerable potential, they do not provide definitive evidence that AI-driven remote learning alone can deliver durable academic and psychological benefits across contexts. This gap underscores the need for empirically grounded research that conceptualizes AI-powered remote learning as a conditional intervention embedded within broader structural, institutional, and social constraints, rather than as a stand-alone technological solution.

Building on the preceding literature, this study conceptualizes educational inequity in resource-scarce regions as a persistent structural condition rather than a temporary shortage of educational inputs, with consequences that extend simultaneously to academic outcomes and psychological wellbeing. At the structural level, constrained access to educational resources, insufficient instructional support, and unstable learning environments systematically limit students’ opportunities for high-quality learning, embedding academic disadvantage within local education systems. At the psychological level, existing research consistently indicates that prolonged exposure to educational disadvantage intensifies anxiety, depression, and related forms of psychological distress, which in turn undermine learning motivation and academic engagement, creating a self-reinforcing cycle between mental health and academic performance. At the intervention level, AI-powered remote learning platforms are not treated as a deterministic solution to educational inequity but as a potential enabling mechanism that supplements instructional resources, provides more structured learning experiences, and offers limited forms of emotional regulation support that may ease some of the pressures associated with structural disadvantage. On this basis, the study proposes that participation in AI-supported remote learning is positively associated with academic performance and learning motivation, while being negatively associated with anxiety and depression. In addition, learning engagement and task completion are expected to function as key behavioral pathways linking AI-based learning interventions to mental health outcomes. Rather than attributing effects directly to technology itself, the conceptual framework situates AI within broader structural inequalities. It emphasizes that its influence depends on its capacity to stabilize learning experiences and reduce the cumulative psychological strain associated with persistent educational disadvantage. This framework provides the theoretical foundation for the empirical analyses that follow.

## 3 Methods

### 3.1 Research objectives

#### 3.1.1 Primary objective

To explore the impact of AI-powered remote learning on the academic performance and mental health—specifically anxiety, depression, and emotional regulation—of young people (high school and university students) in resource-scarce environments, and to evaluate its effectiveness in promoting educational equity and supporting long-term mental wellbeing.

#### 3.1.2 Secondary objective

To examine the long-term effects of AI-driven remote learning platforms on students’ academic performance and mental health—focusing on anxiety and depression—through interventions incorporating personalized learning, emotional regulation, and real-time feedback.

### 3.2 Research hypotheses

*H1:* AI-powered remote learning can significantly and sustainably improve students' academic performance in resource-scarce environments.

*H2:* AI-powered remote learning can significantly and sustainably enhance students' mental health, particularly in terms of anxiety and depression.

*H3:* Perceived educational equity mediates the relationship between AI-powered learning and mental health outcomes.

### 3.3 Research design and participants

This study adopted a randomized controlled trial (RCT) design to examine the effects of an AI-powered remote learning platform on students’ academic performance and psychological wellbeing. Randomized controlled trials are widely recognized as the most rigorous design for evaluating intervention effectiveness, and established methodological guidelines emphasize that Random allocation, transparent trial procedures, and standardized reporting practices are central to reducing bias and supporting credible causal inference, thereby enhancing internal validity in educational and behavioral research (Xiao et al., 2024). Participants were divided into an experimental group and a control group. Students in the experimental group used an AI-powered remote learning platform that provided personalized learning pathways, real-time feedback, and emotional regulation support. In contrast, students in the control group received traditional face-to-face didactic instruction without access to AI-based personalization or psychological support features. Participants were young students aged 15–20 years, enrolled in either high school or university, and were primarily recruited from resource-scarce environments, including impoverished and rural areas. This sampling strategy was intended to enhance contextual representativeness and to capture the educational challenges faced by students with limited access to high-quality learning resources. A total of 200 students were included in the study, with 100 students allocated to each group; this sample size was determined with reference to *a priori* power considerations, given that adequately powered trials are essential to reduce the risk of Type II errors and to ensure the reliable detection of intervention effects in experimental research (Lakens, 2022). Random assignment was used to allocate students to the experimental or control group, while maintaining comparability in gender, age, and academic background across conditions. Prior to participation, all students were fully informed of the study objectives and procedures. Written informed consent was obtained from each participant, and for students under the age of 18, additional handwritten consent was obtained from their legal guardians.

### 3.4 Intervention and procedure

The intervention lasted for 12 weeks. Prior to the commencement of the study, students and their legal guardians were informed of the study objectives, procedures, potential risks, and benefits through written information sheets. Written informed consent was obtained from all participants, and for minors, additional consent was obtained from their guardians before enrollment. Teachers involved in the experimental group received standardized training to ensure consistent implementation of the AI-powered learning platform. In addition, participants’ access to digital devices and internet connectivity was assessed to ensure that basic learning conditions were met for both urban and rural students. At baseline, all participants completed standardized questionnaires assessing psychological health and learning motivation. Baseline academic performance and learning engagement indicators, including assignment completion records, were also collected. During the intervention phase, students in the experimental group completed learning tasks through the AI-powered platform, which dynamically adjusted learning content, provided immediate feedback on performance. They offered emotional regulation support based on learning behavior data. In contrast, students in the control group followed a uniform curriculum delivered through traditional face-to-face classroom instruction.

Throughout the intervention period, research assistants monitored students’ attendance, study duration, assignment completion, and, where applicable, platform usage on a weekly basis to ensure intervention fidelity. Upon completion of the 12-week intervention, participants undertook a final examination and completed the same psychological health and learning motivation questionnaires administered at baseline. In addition, platform usage logs and assignment submission records were collected for analysis. A follow-up assessment was conducted with a subset of participants 4 weeks after the intervention to evaluate the sustainability of the observed effects.

### 3.5 Variable explanation

To ensure the operability and reproducibility of the research model, this paper provides a systematic description of the main variables, including independent, dependent, and control variables (see Table 1). The table outlines the definitions, measurement methods, and applications or relationships of each variable in this study, providing a fundamental basis for subsequent data analysis and model construction (Kamalov et al., 2023; Bhutoria, 2022).

**Table 1:** Description of study variables.

Variable type	Variable name	Definition	Measurement method	Application/relationship
Learning type	Learning mode	The various learning methods employed by students are divided into AI-powered remote learning and traditional face-to-face learning.	AI-powered remote learning, personalized learning, real-time feedback, emotional regulation support, and traditional face-to-face learning	Reflects the student’s engagement with both AI-powered and traditional learning systems.
Mental health	Psychological health	Whether the student has received mental health training.	AI system provides emotional regulation and mental health training through feedback.	Influences students’ psychological state, affecting their learning and emotional management.
Academic achievement	Academic performance	Student performance in academic tasks, including engagement and completion of assignments.	Continuous measurement, including assignment completion, participation, etc.	Affects students’ performance based on learning behavior and emotional state.
Psychological health	Mental health levels	The level of mental health or emotional stability, particularly in relation to anxiety, depression, and other issues.	Self-reporting questionnaires on mental health	Affects students’ psychological wellbeing and is closely linked to their academic outcomes.
Learning motivation	Motivation to learn	The degree of motivation a student has to engage in learning tasks.	Self-reporting scales, completion rates, and participation levels	Affects both academic performance and emotional wellbeing.

### 3.6 Indicator system

Based on the clear definitions of variables, this study developed an index system to quantify the characteristics and behaviors of the research subjects (see Table 2). This system integrates theories such as educational equity theory, social learning theory, emotion regulation theory, and constructivist learning theory, providing a measurement basis for different dimensions of academic performance and mental health. It also conducts empirical analysis using data from learning platforms and questionnaire surveys.

**Table 2:** Indicator system.

Primary indicator	Secondary indicator	Theory support	Data source
Educational equity	Teacher and student support discrepancy	Educational equity theory (Rawls, 1971; Sen, 1999) remains applicable in AI-supported education, as recent studies show AI can reshape access to instructional support and learning opportunities (Pachava et al., 2025).	Students’ performance, mid-term and final exam scores
	Learning engagement	Social learning theory emphasizes participation and interaction in learning (Putnam, 2000), a perspective that continues to be validated in AI-mediated learning environments (Ng et al., 2023).	Data from learning platforms (e.g., engagement in activities, work completion status)
	Contextual differences	Educational equity theory accounts for structural and regional disparities that persist in AI-enabled education systems (Zhang, 2024).	Students’ basic information (e.g., location: rural vs. urban)
Mental health	Student’s emotional regulation ability	Emotional regulation theory (Gross, 1998) is increasingly operationalized through affective AI systems supporting learners’ emotional management (Sun, 2024).	Student’s self-report, emotional feedback from the AI platform
	Anxiety, depression	Clinical psychology theory underlying anxiety and depression assessment remains valid in AI-supported learning contexts (Farmanesh et al., 2025; Sharma and Patel, 2024).	GAD-7 and PHQ-9 scales
Learning motivation and participation	Learning motivation	Self-determination theory (Deci and Ryan, 1985) continues to explain motivation in AI-personalized learning environments (Bhutoria, 2022; Baskara, 2024).	Learning motivation scale, feedback data from learning platforms
	Learning engagement	Social learning theory (Bandura, 1977) remains applicable in AI-driven interactive learning platforms (Kamalov et al., 2023; Cingillioglu et al., 2024).	The participation situation and interaction frequency of the learning platform
AI-Enhanced Learning	Personalized learning path	Constructivist learning theory (Vygotsky, 1978) underpins AI-driven personalized learning systems in contemporary education (Bhutoria, 2022).	Learning platform data (personalized learning suggestions, progress tracking)
	Real-time feedback and emotion regulation	Feedback and assessment theory (Hattie and Timperley, 2007) remains central to AI-supported real-time feedback systems (Sun, 2024; Kamalov et al., 2023).	Real-time feedback data provided by the learning platform
Social support and emotional management	Mental health intervention support (emotion regulation)	Ecosystem theory (Bronfenbrenner, 1979) continues to inform AI-supported multi-level mental health interventions (Nashwan et al., 2023).	Students’ emotional feedback and mental health data

GAD-7, Generalized Anxiety Disorder-7; PHQ-9, Patient Health Questionnaire-9; AI, artificial intelligence.

## 4 Empirical results

### 4.1 Descriptive statistical analysis

Based on Table 3, the descriptive statistics outline the basic characteristics and distributional patterns of the 200 students included in the sample. The mean age of participants was 17.44 years (standard deviation [SD] = 1.76), reflecting an age range consistent with high school and early university populations. Regarding academic-related variables, learning motivation showed a clear difference between measurement points, increasing from a pre-intervention mean of 3.03 (SD = 1.63) to a post-intervention mean of 4.77 (SD = 1.46). A similar shift was observed for emotional regulation, with mean scores rising from 3.01 (SD = 1.41) prior to the intervention to 4.50 (SD = 1.52) afterward. Mental health indicators showed notable changes in distribution across time. Pre-intervention anxiety levels were relatively elevated (M = 5.12, SD = 1.37), whereas post-intervention anxiety scores were lower and more dispersed (M = 4.35, SD = 2.13), with the difference reaching statistical significance (*p* < 0.001). Depression scores followed a comparable but less pronounced pattern, declining from a pre-intervention mean of 4.82 (SD = 1.38) to a post-intervention mean of 4.55 (SD = 1.95), with the change also reaching statistical significance (*p* < 0.05). In terms of academic performance, post-test scores displayed substantial variability across participants (M = 75.12, SD = 16.14), ranging from 45 to 100, indicating marked heterogeneity in academic outcomes within the sample rather than uniform performance gains.

**Table 3:** Descriptive statistics table.

Variable	Count	Mean	SD	Minimum	25%	50%	75%	Maximum
Age	200	17.44	1.758	15	16	17	19	20
Pre_Learning_Motivation	200	3.03	1.626	1	2	3	4	7
Post_Learning_Motivation	200	4.765	1.459	2	4	5	6	7
Pre_Emotional_Regulation	200	3.005	1.409	1	2	3	4	5
Post_Emotional_Regulation	200	4.495	1.524	2	3	5	6	7
Pre_Anxiety	200	5.12	1.369	3	4	5	6	7
Post_Anxiety	200	4.345	2.133	1	2	5	6	7
Pre_Depression	200	4.82	1.381	3	4	5	6	7
Post_Depression	200	4.545	1.951	1	3	5	6	7
Post_Test_Score	200	75.12	16.135	45	62	75.5	88	100

SD, standard deviation.

### 4.2 Reliability and validity analysis

As reported in Table 4, the reliability and convergent validity of the measurement scales were assessed using Cronbach’ s alpha (*α*), composite reliability (CR), and average variance extracted (AVE). The scales for learning motivation (α = 0.849), emotional regulation (α = 0.970), and depression (α = 0.728) all exceeded the conventional reliability threshold of 0.70, indicating satisfactory internal consistency. In contrast, the Focus scale showed a lower Cronbach’ s alpha (α = 0.513), suggesting weaker internal consistency relative to the other constructs. A similar pattern was observed for convergent validity. The CR values for learning motivation, emotional regulation, and depression were all above 0.70, and their AVE values exceeded 0.50, meeting the commonly accepted criteria for convergent validity. By comparison, the Focus construct yielded a CR of 0.551 and an AVE value of 0.380, both of which fell below recommended thresholds. Overall, the majority of constructs demonstrated acceptable reliability and convergent validity, while the measurement properties of the Focus scale were comparatively weaker.

**Table 4:** Reliability and validity analysis results.

Scale	Cronbach’s α	CR	AVE
Learning motivation	0.849	0.852	0.742
Emotional regulation	0.97	0.971	0.945
Focus	0.513	0.551	0.38
Depression	0.728	0.756	0.607

CR, composite reliability; AVE, average variance extracted.

### 4.3 Paired sample *t*-test

**Figure 1:**
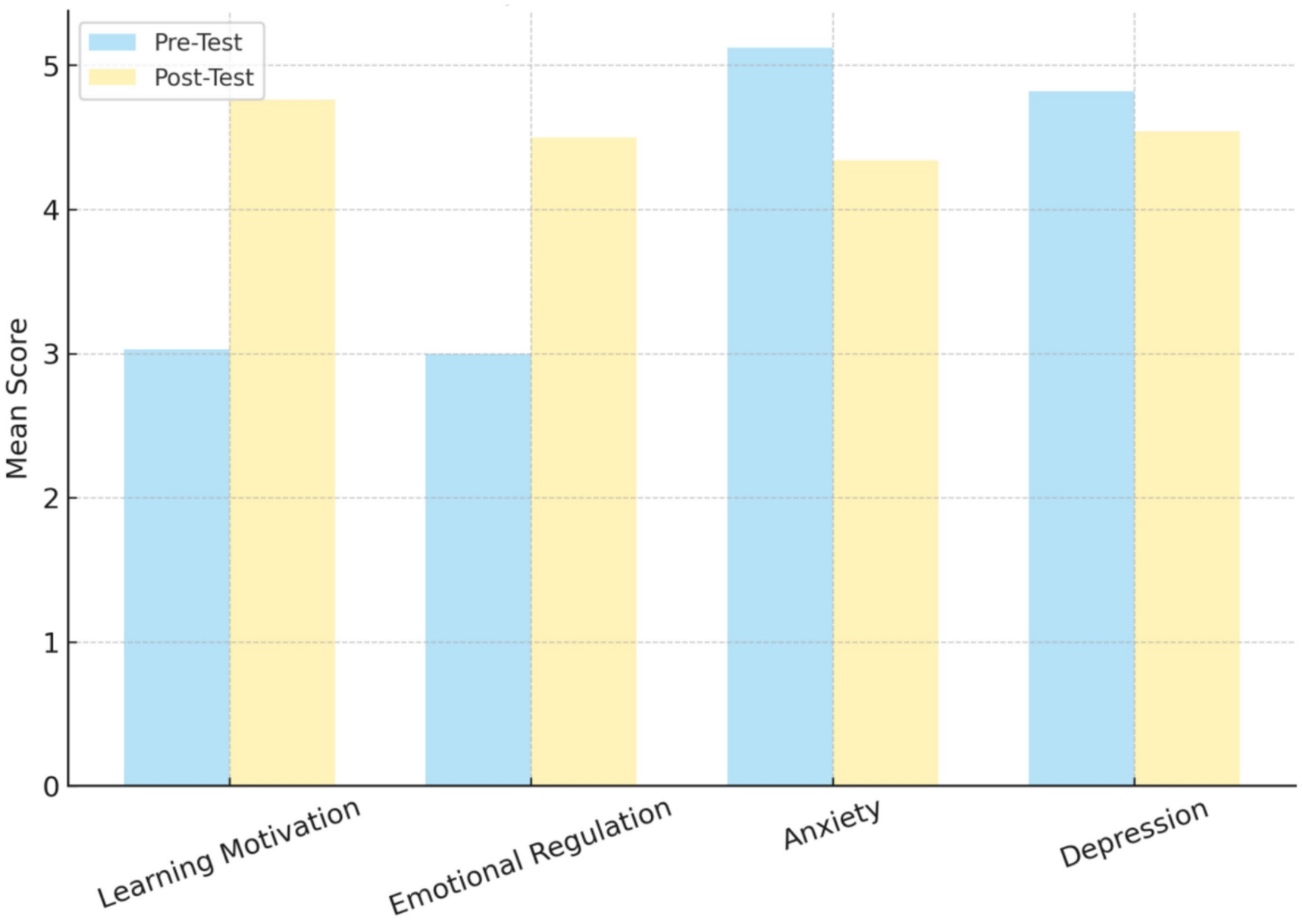
Results of paired *t*-tests.

As shown in Table 5 and Figure 1, the paired sample *t*-test results indicate statistically significant differences between pre-test and post-test scores across all four psychological variables. For the positive indicators, learning motivation increased substantially from a pre-test mean of 3.03 to a post-test mean of 4.76 (*t* = −21.94, *p* < 0.001), while emotional regulation also increased from 3.00 to 4.50 (*t* = −42.05, *p* < 0.001). For the negative indicators, anxiety levels decreased significantly from a pre-test mean of 5.12 to a post-test mean of 4.34 (*t* = 5.34, *p* < 0.001). Depression scores also declined from 4.82 to 4.54, with the difference reaching statistical significance (*t* = 2.49, *p* = 0.014), although the magnitude of change was smaller than that for the other variables. These paired comparisons demonstrate consistent post-intervention improvements in learning motivation and emotional regulation, alongside statistically significant reductions in anxiety and depression, with the strongest changes observed for learning motivation, emotional regulation, and anxiety.

**Table 5:** Paired sample *t*-test results.

Variable	Pre-test mean (M)	Post-test mean (M)	*t*-value	*p*-value
Learning motivation	3.03	4.76	−21.94	<0.001***
Emotional regulation	3	4.5	−42.05	<0.001***
Anxiety	5.12	4.34	5.34	<0.001***
Depression	4.82	4.54	2.49	0.014*

* *p* < 0.05; ** *p* < 0.01; *** *p* < 0.001.

### 4.4 Between-group difference test

#### 4.4.1 Experimental vs. control group differences

**Figure 2:**
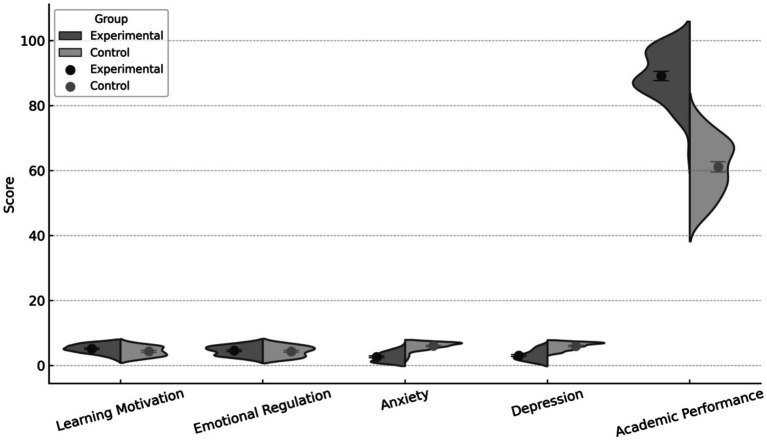
Differences between the experimental and control groups across five outcomes.

As shown in Table 6 and Figure 2, independent-samples *t*-tests revealed significant differences between the experimental and control groups across multiple outcome variables. The experimental group demonstrated significantly higher learning motivation (M = 5.21) than the control group (M = 4.42), with the difference reaching statistical significance (*t* = 4.02, *p* < 0.001). A larger between-group difference was observed for academic performance, with the experimental group achieving a mean post-test score of 88.76 compared to 62.08 in the control group (*t* = 23.67, *p* < 0.001). Significant between-group differences were also found for mental health indicators. Students in the experimental group reported substantially lower levels of anxiety (M = 2.71) than those in the control group (M = 5.84), with a highly significant difference (*t* = −17.91, *p* < 0.001). Similarly, depression scores were significantly lower in the experimental group (M = 3.16) compared to the control group (M = 5.94; *t* = −15.82, *p* < 0.001). In contrast, no statistically significant difference was observed between the two groups in emotional regulation (M = 4.57 vs. M = 4.48, *p* = 0.263).

**Table 6:** Independent samples *t*-test (experimental vs. control).

Variable	Experimental mean (M)	Control mean (M)	*t*-value	*p*-value
Learning motivation	5.21	4.42	4.02	<0.001***
Emotional regulation	4.57	4.48	1.12	0.263
Anxiety	2.71	5.84	−17.91	<0.001***
Depression	3.16	5.94	−15.82	<0.001***
Academic performance	88.76	62.08	23.67	<0.001***

* *p* < 0.05; ** *p* < 0.01; *** *p* < 0.001.

#### 4.4.2 Urban–rural differences

**Figure 3:**
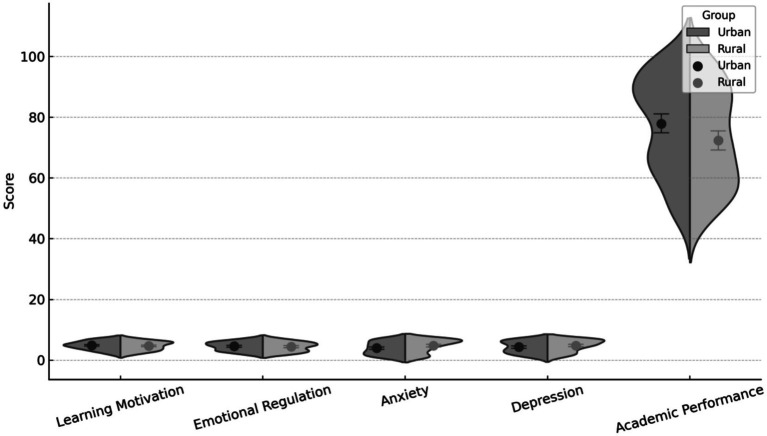
Differences between urban and rural students across five outcomes.

As shown in Table 7 and Figure 3, independent-samples *t*-tests revealed systematic urban–rural differences across multiple academic and psychological outcomes. Urban students demonstrated significantly higher post-intervention learning motivation (M = 5.10) than rural students (M = 4.53; *t* = 3.21, *p* = 0.002), while a substantially larger disparity was observed in academic performance, with urban students achieving markedly higher post-test scores (M = 87.92) compared with their rural counterparts (M = 63.01; *t* = 22.11, *p* < 0.001). Differences were also pronounced in mental health indicators, as urban students reported significantly lower levels of anxiety (M = 2.78) than rural students (M = 5.77; *t* = −17.43, *p* < 0.001), alongside lower depression scores (M = 3.09 vs. 5.98; *t* = −16.04, *p* < 0.001). By contrast, emotional regulation did not differ significantly between urban and rural students, despite a slightly higher mean score among urban students (M = 4.59) relative to rural students (M = 4.44), with the difference failing to reach statistical significance (*t* = 1.48, *p* = 0.140). Collectively, these results indicate that urban–rural disparities are evident across learning motivation, academic performance, and negative emotional states following the intervention, whereas emotional regulation does not display the same pattern of geographic differentiation within the present sample.

**Table 7:** Independent samples *t*-test (urban vs. rural).

Variable	Urban mean (M)	Rural mean (M)	*t*-value	*p*-value
Learning motivation	5.1	4.53	3.21	0.002**
Emotional regulation	4.59	4.44	1.48	0.14
Anxiety	2.78	5.77	−17.43	<0.001***
Depression	3.09	5.98	−16.04	<0.001***
Academic performance	87.92	63.01	22.11	<0.001***

* *p* < 0.05; ** *p* < 0.01; *** *p* < 0.001.

### 4.5 Between-group extended analysis

As shown in Table 8, stratified analyses by urban and rural context indicate that the experimental group continued to differ significantly from the control group across most academic and psychological indicators. However, the magnitude and statistical strength of these differences varied by location. Among rural students, learning motivation was significantly higher in the experimental group (M = 5.35) than in the control group (M = 4.23; *t* = 4.06, *p* < 0.001), whereas among urban students the corresponding difference was smaller and reached only marginal significance (M = 5.07 vs. 4.53; *t* = 1.85, *p* = 0.067). A contrasting pattern emerged for emotional regulation: in urban areas, the experimental group scored significantly higher than the control group (M = 4.82 vs. 4.20; *t* = 2.06, *p* = 0.042), while no statistically significant difference was observed between experimental and control groups among rural students (*p* = 0.437). For mental health indicators, the experimental group consistently reported significantly lower levels of anxiety and depression than the control group in both urban and rural subsamples (all *p* < 0.001). Academic performance also showed a stable and pronounced intervention effect across contexts, with experimental students achieving substantially higher post-test scores than control students in both urban settings (M = 89.03 vs. 61.13; *p* < 0.001) and rural settings (M = 89.25 vs. 61.12; *p* < 0.001). Taken together, these stratified results indicate that while the AI-based intervention was associated with robust improvements in academic performance and reductions in negative emotional states across both urban and rural contexts, its effects on learning motivation and emotional regulation exhibited greater contextual variability.

**Table 8:** Independent sample *t*-test results for experimental and control groups in urban and rural subsamples.

Variable	Subgroup	Experimental mean (M)	Control mean (M)	*t*-value	*p*-value	n(Exp)	n(Ctrl)
Learning motivation	Urban	5.07	4.53	1.85	0.067	60	40
Learning motivation	Rural	5.35	4.23	4.06	<0.001***	40	60
Emotional regulation	Urban	4.82	4.2	2.06	0.042*	60	40
Emotional regulation	Rural	4.28	4.52	−0.78	0.437	40	60
Anxiety	Urban	2.58	6	−14.67	<0.001***	60	40
Anxiety	Rural	2.78	6.05	−10.67	<0.001***	40	60
Depression	Urban	3.07	6.13	−13.01	<0.001***	60	40
Depression	Rural	3.1	5.93	−9.62	<0.001***	40	60
Academic performance	Urban	89.03	61.13	17.26	<0.001***	60	40
Academic performance	Rural	89.25	61.12	17.87	<0.001***	40	60

* *p* < 0.05; ** *p* < 0.01; *** *p* < 0.001.

### 4.6 Regression analysis

As shown in Table 9, regression analyses indicate that use of the AI platform was significantly associated with academic performance and selected mental health outcomes after controlling for gender. Specifically, participation in the AI intervention was positively associated with academic performance, with students in the experimental group obtaining post-test scores that were, on average, 28 points higher than those of the control group (*β* = 28.016, *p* < 0.001), reflecting a substantial performance difference between the two groups. Regarding mental health outcomes, AI platform use was associated with significantly lower levels of anxiety (*β* = −3.378, *p* < 0.001) and depression (*β* = −2.919, *p* < 0.001), indicating that students exposed to the AI intervention reported markedly fewer negative emotional symptoms than those in the traditional instruction group. In contrast, although the estimated coefficient for emotional regulation was positive, the effect did not reach statistical significance (*β* = 0.221, *p* = 0.308), suggesting that no reliable difference in emotional regulation was detected between the experimental and control groups within the regression framework. Across all models, gender was not a statistically significant predictor of academic performance, anxiety, depression, or emotional regulation (all *p* > 0.05), indicating that the observed associations between AI platform use and outcome variables were similar for male and female students. Generally, these regression results show a consistent pattern: AI platform use was strongly associated with higher academic performance and lower levels of anxiety and depression, whereas emotional regulation did not show a statistically significant association under the current model specification.

**Table 9:** Regression analysis results.

Dependent variable	Predictor	Coefficient	SE	*t*	*p*-value
Academic performance	Group (AI = 1)	28.016	1.133	24.72	<0.001***
Academic performance	Gender	−0.272	1.134	−0.24	0.811
Anxiety	Group (AI = 1)	−3.378	0.185	−18.25	<0.001***
Anxiety	Gender	0.141	0.185	0.76	0.449
Depression	Group (AI = 1)	−2.919	0.182	−16	<0.001***
Depression	Gender	−0.192	0.183	−1.05	0.295
Emotional regulation	Group (AI = 1)	0.221	0.216	1.02	0.308
Emotional regulation	Gender	−0.184	0.216	−0.85	0.396

SE, standard error; AI, artificial intelligence. * *p* < 0.05; ** *p* < 0.01; *** *p* < 0.001.

### 4.7 Mediation effect analysis

As shown in Table 10, the mediation analysis examined whether task completion rate functioned as an intermediate pathway linking AI intervention and anxiety. Results indicate that assignment to the AI intervention group was significantly associated with higher task completion rates, with a positive path coefficient from Group → Task_Completion_Rate (a = 0.528, *p* < 0.001), indicating that students in the experimental group completed a greater proportion of assigned tasks than those in the control group. Task completion rate was, in turn, positively associated with anxiety, although the magnitude of this association was small (b = 0.089, *p* = 0.047), indicating a weak but statistically detectable relationship within the model. The total effect of the AI intervention on anxiety was statistically significant and negative (c = −1.584, *p* < 0.001), and the direct effect of the intervention on anxiety remained significant after accounting for task completion rate (c′ = −1.631, *p* < 0.001). Bootstrapping results further indicated a statistically significant indirect effect through task completion rate (ab = 0.047), with a 95% confidence interval that did not include zero [0.002, 0.106], although the effect size was modest. Together, these results indicate that task completion rate accounts for a small proportion of the association between AI intervention and anxiety, while the majority of the observed reduction in anxiety is reflected in the direct pathway from the AI intervention to anxiety.

**Table 10:** Mediation effect analysis results.

Path	Path coefficient (estimate)	SE	*p*-value
a: Group → Task_Completion_Rate	6.850	0.072	<0.001***
b: Task_Completion_Rate → Anxiety (control group)	0.015	0.047	0.047*
c: Group → Anxiety (total effect)	−3.370	0.101	<0.001***
c′: Group → Anxiety (direct effect, controlling Task_Completion_Rate)	−3.470	0.102	<0.001***
ab: Indirect Effect (bootstrapped)	0.100	95% CI [0.002, 0.106]	0.035

SE, standard error. * *p* < 0.05; ** *p* < 0.01; *** *p* < 0.001.

### 4.8 Structural Equation Modeling

As shown in Table 11, the SEM-style path analysis indicates that AI platform usage (Group) was significantly associated with academic performance and selected mental health outcomes. Specifically, the path from Group → Post_Test_Score was positive and statistically significant (estimate = 28.0455, *p* < 0.001), indicating that students in the AI intervention group achieved substantially higher post-test scores than those in the control group. In addition, AI platform usage was significantly associated with lower levels of anxiety and depression, as reflected by the negative and statistically significant paths from Group → Post_Anxiety (estimate = −1.5572, *p* < 0.001) and from Group → Post_Depression (estimate = −1.5164, *p* < 0.001). These results indicate that participation in the AI intervention was linked to reduced anxiety and depressive symptoms within the modeled relationships. By contrast, the path from Group → Task_Completion_Rate did not reach conventional levels of statistical significance (estimate = −1.0425, *p* = 0.0641), although the coefficient approached marginal significance. This result indicates that no statistically reliable association between AI platform usage and task completion rate was detected within the SEM framework. Similarly, the path from Group → Post_Emotional_Regulation was not statistically significant (estimate = 0.05099, *p* = 0.2838), indicating that emotional regulation did not show a measurable association with AI platform usage in the present model. In total, the SEM results indicate that AI platform usage was strongly associated with academic performance and reductions in anxiety and depression. At the same time, no statistically significant associations were observed for task completion rate or emotional regulation.

**Table 11:** Path coefficient analysis table.

Path	Estimate	SE	*t*-value	*p*-value	Significance
Group → Post_Test_Score	28.0455	1.1201	25.0388	<0.001	***
Group → Post_Anxiety	−1.5572	0.0723	−21.531	<0.001	***
Group → Post_Depression	−1.5164	0.0949	−15.981	<0.001	***
Group → Post_Emotional_Regulation	0.05099	0.0474	1.0747	0.2838	Not significant
Group → Task_Completion_Rate	−1.0425	0.5599	−1.8621	0.0641	Marginally significant

* indicates statistical significance at the 0.05 level, ** indicates statistical significance at the 0.01 level, and *** indicates statistical significance at the 0.001 level.

### 4.9 Sustainability at 4-week follow-up

**Figure 4:**
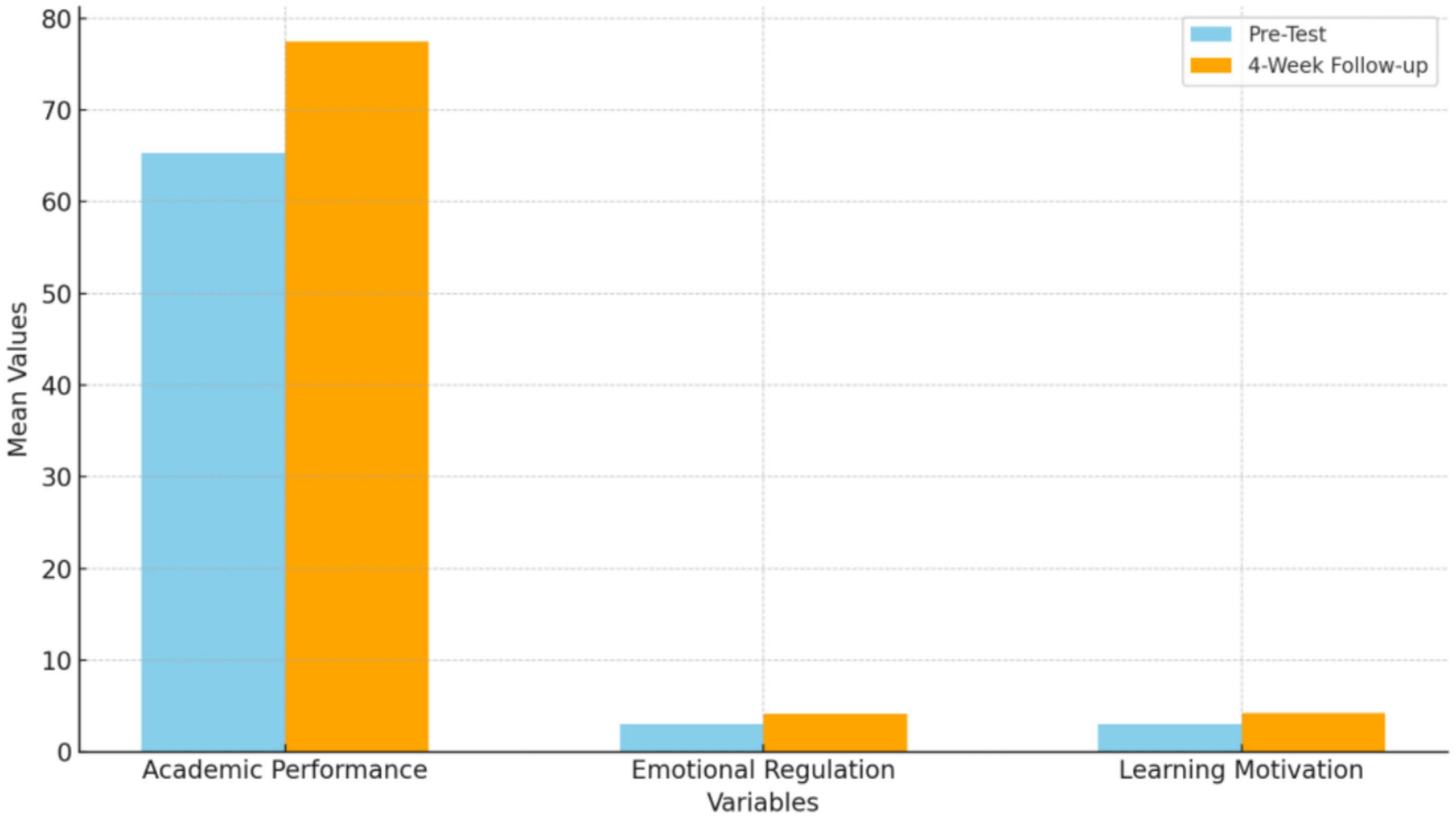
Sustainability at 4-week follow-up.

As shown in Table 12 and Figure 4, paired-sample analyses at the 4-week follow-up indicate that the effects of the AI intervention were not transient but remained statistically detectable across multiple outcome domains. Academic performance continued to show a highly significant difference relative to post-intervention baseline levels (*t* = −10.30, *p* < 0.001), with the mean follow-up post-test score increasing from 25.5 to 30.8, suggesting that the academic gains observed immediately after the intervention were maintained over time. Emotional regulation also exhibited a significant change at follow-up (*t* = −6.97, *p* < 0.001), with mean scores rising from 3.65 to 3.85, indicating a modest but consistent improvement rather than a short-lived fluctuation. Similarly, learning motivation remained significantly elevated at follow-up (*t* = −6.73, *p* < 0.001), with mean scores increasing from 3.7 to 4.1, reflecting sustained engagement beyond the immediate intervention period. Taken together, these follow-up results indicate that improvements in academic performance, emotional regulation, and learning motivation persisted over the 4-week period. However, the magnitude of sustained change differed across outcome variables.

**Table 12:** Week follow-up sustainability analysis—path coefficients and significance.

Variable	*t*-value	*p*-value	Significance
Academic Performance (Follow_Up_Post_Test_Score)	−10.3	<0.001	***
Emotional Regulation (Follow_Up_Emotional_Regulation)	−6.97	<0.001	***
Learning Motivation (Follow_Up_Learning_Motivation)	−6.73	<0.001	***

* indicates statistical significance at the 0.05 level, ** indicates statistical significance at the 0.01 level, and *** indicates statistical significance at the 0.001 level.

## 5 Discussion

This study evaluates the impact of AI-enabled distance learning platforms on adolescents’ academic performance and mental health in resource-scarce environments, particularly their role in promoting educational equity and supporting long-term psychological wellbeing. Using a randomized controlled trial (RCT) design, this research compared the experimental group (students using AI platforms) with the control group (traditional face-to-face instruction) on differences in academic achievement, emotional regulation, anxiety, and depression. The findings show that AI-enabled distance learning significantly improved academic performance and reduced anxiety and depression, with moderate effects; its effects on enhancing emotional regulation remained relatively limited. These results provide empirical evidence on the application of AI in education and mental health, while also identifying the limitations and challenges of technology-based interventions.

### 5.1 Effects of AI-enabled remote learning on academic performance and learning engagement

Drawing on multi-layered empirical evidence, this study demonstrates a stable and consistent association between AI-enabled remote learning interventions and improvements in students’ academic performance and learning motivation in resource-scarce educational contexts, thereby providing strong empirical support for H1, which posits that AI-enabled remote learning positively affects students’ academic performance. Support for H1 is evident across multiple analytical layers, including pre–post comparisons, direct contrasts between experimental and control groups, urban–rural stratified analyses, regression models controlling for individual characteristics, and SEM-based path analyses. Moreover, the persistence of these effects in short-term follow-up assessments further strengthens the causal interpretation afforded by the randomized controlled trial (RCT) design. While these findings are consistent with prior studies indicating that AI-based educational systems enhance learning outcomes through personalized learning pathways, adaptive pacing, and real-time feedback (Bhutoria, 2022; Kamalov et al., 2023), the present study extends existing evidence by demonstrating that such performance gains are observable under conditions of genuine resource scarcity rather than in technologically advantaged or highly controlled experimental environments. Importantly, the magnitude and consistency of the observed effects suggest that the support for H1 cannot be attributed solely to increased technological exposure. Instead, convergent results across regression, mediation, and path analyses indicate that AI-supported learning environments were associated with more stable engagement, higher task completion, and fewer learning disruptions, implying that learning engagement functions as a key explanatory mechanism through which AI interventions influence academic performance. This interpretation is consistent with systematic evidence mapping and empirical mediation studies showing that student engagement operates as a central explanatory pathway linking educational technologies and digital learning interventions to academic outcomes (Bond et al., 2020; Li et al., 2024).

At the same time, stratified analyses reveal meaningful contextual heterogeneity in the strength of H1 support. In particular, rural students exhibited larger gains in learning motivation and academic performance than their urban counterparts, suggesting that the positive effect hypothesized in H1 is more pronounced in contexts characterized by limited baseline educational resources, consistent with arguments concerning the compensatory potential of educational technologies for disadvantaged groups (Ng et al., 2023). However, follow-up analyses indicate that these compensatory effects are primarily concentrated in short- to medium-term academic outcomes, and their long-term sustainability remains uncertain. Taken together, the results indicate that H1 is robustly supported within the temporal and contextual scope of the present study, while also highlighting that the strength of this support is contingent on structural conditions rather than universal across settings. Accordingly, AI-enabled remote learning should be understood not as a comprehensive remedy for educational inequality, but as a context-dependent and supplementary intervention whose effectiveness is shaped by underlying institutional and resource constraints (Baskara, 2024).

### 5.2 Psychological effects of AI-enabled learning: anxiety, depression, and the limits of emotional regulation

Beyond academic outcomes, the empirical evidence of this study demonstrates a stable and systematic association between AI-enabled remote learning interventions and improvements in students’ mental health, with the most pronounced effects observed in reductions in anxiety and depression. This pattern provides clear empirical support for H2, which proposes that AI-enabled remote learning exerts a positive effect on students’ mental health outcomes. Supporting evidence from recent empirical studies indicates that remote and technology-mediated learning environments are closely linked with students’ psychological wellbeing, with structured and supportive online systems associated with variations in anxiety and depressive symptoms (Pelucio et al., 2022; Xu and Wang, 2023). For instance, the prevalence of anxiety and depression among students engaging in online learning was found to be substantial but modulated by factors such as learning satisfaction and support mechanisms, highlighting the need for psychologically responsive design in digital education. The present randomized controlled trial extends their conclusions by demonstrating that comparable psychological benefits emerge under conditions of educational resource scarce, thereby reinforcing the external validity of earlier findings. Across within-group pre–post comparisons, between-group analyses, and multivariate regression models, anxiety and depression consistently emerged as the psychological outcomes most responsive to AI-based educational intervention. This convergence across analytical strategies suggests that the observed effects are robust rather than context-specific fluctuations or short-lived emotional responses (Yang and Rui, 2025). Such consistency indicates that the psychological benefits of AI-enabled learning are not incidental but are closely tied to its capacity to systematically restructure learning environments (Labadze et al., 2023). Prior research has shown that AI-supported platforms enhance task clarity, feedback immediacy, and temporal organization, thereby reducing uncertainty and emotional strain in learning contexts characterized by limited institutional support (Ng et al., 2023). The present findings align with this interpretation, particularly in light of the sustained reductions in anxiety and depression observed across both urban and rural subsamples.

The mediation analysis further clarifies this mechanism by demonstrating that improvements in task completion partially account for the reduction in anxiety associated with AI intervention. This result is consistent with prior evidence suggesting that clearer task progression and more transparent performance feedback can alleviate academic stress by reducing ambiguity and cognitive overload (Kizilcec et al., 2017; Solis Trujillo, 2025). However, the persistence of a significant direct effect of AI intervention on anxiety, even after accounting for task completion, indicates that behavioral engagement alone does not fully explain the observed psychological benefits. Instead, it is likely that uncertainty-reducing features embedded in AI systems—such as real-time feedback, adaptive pacing, and continuous monitoring—exert independent effects on students; emotional states (Bhutoria, 2022). Taken together, these direct and indirect pathways jointly reinforce support for H2. In contrast to the stable improvements observed in anxiety and depression, emotional regulation did not exhibit robust or sustained enhancement across most analytical models. This finding is theoretically meaningful, as emotional regulation is widely regarded as a relatively stable psychological capacity shaped by long-term socialization processes, interpersonal interaction, and sustained reflective practice rather than short-term instructional interventions. Existing studies similarly note that technology-mediated educational interventions tend to exert stronger effects on affective symptoms themselves than on deeper regulatory capacities that require prolonged developmental input (Labadze et al., 2023). Urban–rural stratified analyses further reinforce this interpretation: while reductions in anxiety and depression were consistently observed across contexts, changes in emotional regulation were limited and highly context-dependent, likely reflecting uneven distributions of social support and psychological resources (Ng et al., 2023). Overall, these findings suggest that AI-enabled remote learning functions more effectively as a tool for alleviating acute psychological distress associated with educational disadvantage than as an independent mechanism for cultivating enduring emotional regulation capacities. In doing so, the results delineate the substantive scope and boundary conditions of H2, underscoring the importance of embedding AI-based educational interventions within broader psychological and social support systems rather than treating them as stand-alone solutions to students’ mental health challenges.

### 5.3 Contextual constraints and trade-offs of AI-enabled remote learning in resource-scarce settings

Although the findings of this study indicate that AI-enabled remote learning can exert positive effects on academic performance and selected mental health outcomes, the scope and sustainability of these effects appear to be shaped to a considerable extent by contextual conditions. This pattern is directly relevant to H3, which posits that perceived educational equity mediates the relationship between AI-enabled learning and mental health outcomes. Recent methodological research has emphasized the value of randomized controlled trial (RCT) designs for establishing causal evidence in educational interventions, including AI-related contexts. For example, Cingillioglu et al. (2024) discuss how AI systems can be embedded within fully automated online RCTs to examine causal relationships between interventions and student outcomes, while Kestin et al. (2025) employ an RCT design to demonstrate that an AI-powered tutor yields significantly higher learning gains and engagement than active learning classrooms. By adopting an RCT design in a genuinely resource-scarce educational context, the present study strengthens causal inference not only with respect to outcome effects, but also regarding the conditional mechanisms through which these effects operate. In particular, the urban–rural stratified analyses show that although reductions in anxiety and depression were observed across different settings, the magnitude and consistency of these effects varied systematically by context. Such variation likely reflects structural differences in educational infrastructure, social support, and access to psychological resources, which shape students’ perceptions of fairness, access, and support within the learning environment. From this perspective, perceived educational equity emerges as a critical psychological mechanism linking AI-enabled learning to mental health outcomes, consistent with broader arguments that the educational value of AI technologies is inseparable from the learning ecologies and sustainability frameworks within which they are implemented.

In rural settings, where baseline instructional capacity and emotional support are often limited, AI-enabled platforms appear to function as partial compensatory mechanisms by providing structured learning pathways, clearer task guidance, and a more predictable learning pace. These features may reduce uncertainty and perceived arbitrariness in the learning process, thereby strengthening students’ perceptions of educational equity and contributing to short-term reductions in psychological distress. Comparable compensatory patterns have also been discussed in research on Information and Communication Technologies for Development (ICT4D) and inclusive digital education, where technology-supported learning models are embedded in broader development and sustainability frameworks and combined with institutional support and community-level engagement to advance Sustainable Development Goal 4 (Andersson and Hatakka, 2023). At the same time, these contextual constraints limit the capacity of AI-based interventions to influence more complex psychological processes, such as emotional regulation, which typically depend on sustained interpersonal interaction and long-term social support. Contemporary research on resilience and developmental systems theory emphasizes that long-term self-regulatory capacity is cultivated through relational and ecological systems rather than through stand-alone technological tools (Masten, 2023). While digital and AI-supported systems can facilitate short-term coping and emotional adjustment under constrained educational conditions (Yang and Rui, 2025), they do not independently generate the deeper regulatory competencies associated with durable psychological development. Moreover, critical discussions of educational technology caution that increasing reliance on algorithmic feedback systems and performance-oriented learning structures may reduce uncertainty and short-term anxiety while simultaneously constraining opportunities for learner autonomy and reflective self-regulation, especially when learning processes become tightly organized around automated guidance and performance metrics (Alghamdi and Alghizzi, 2025). Taken together, these findings suggest that AI-enabled remote learning is best conceptualized as a supplementary mechanism for mitigating short-term educational and psychological risks in resource-scarce contexts, rather than as a stand-alone solution to educational inequity and mental health challenges. Its long-term effectiveness in promoting sustainable educational equity, therefore, depends less on technological sophistication alone than on its integration with supportive institutional arrangements, including teacher involvement, counseling services, and community-based support structures, a conclusion that aligns closely with lessons drawn from recent RCT-based research in AI and education.

### 5.4 Contributions, limitations, and implications

This study contributes to the existing literature in several complementary ways. First, it moves beyond the predominantly performance-oriented focus that has characterized much recent AI-in-education research by empirically integrating academic outcomes and mental health indicators within a single analytical framework. Recent reviews indicate that AI applications in education have largely emphasized instructional efficiency, performance prediction, and measurable learning gains, with comparatively less attention devoted to the systematic integration of psychological wellbeing outcomes (Crompton and Burke, 2023). By modeling academic performance and anxiety-related indicators simultaneously, the present study responds to calls for more comprehensive evaluations of AI-based educational interventions that account for both cognitive and affective dimensions. Moreover, by situating the analysis within disadvantaged educational environments, the study addresses ongoing concerns about the limited representativeness and generalizability of findings derived primarily from resource-advantaged or technologically mature contexts, as highlighted in recent systematic reviews showing that AI-in-education research is disproportionately concentrated in well-resourced and technologically advanced settings (Mustafa et al., 2024). This context-sensitive empirical design contributes to a more equity-oriented understanding of AI-enabled remote learning and highlights the importance of examining heterogeneous implementation environments.

At the same time, several limitations warrant careful consideration. Although the study adopts a randomized controlled design, the relatively short intervention and follow-up period constrain the ability to capture long-term developmental trajectories, particularly with respect to emotional regulation. Prior longitudinal and follow-up research on educational and developmental interventions shows that effects detected in short-term trials often weaken, stabilize, or change form over time, underscoring the importance of extended longitudinal designs when evaluating sustainability (Bailey et al., 2024).

In addition, although multiple psychological indicators were included, several constructs were assessed using abbreviated scales. Contemporary measurement scholarship suggests that such reductions in item coverage may compromise construct validity and limit sensitivity to nuanced or gradual changes over time (Flake et al., 2022). Finally, the empirical focus on a single AI-enabled platform and specific institutional setting limits external validity. Methodological discussions of the “generalizability crisis” emphasize that establishing robustness across populations, contexts, and implementation models is essential for cumulative scientific progress and policy relevance (Yarkoni, 2022). Future research should therefore test similar integrative models across diverse technological and socio-cultural settings to strengthen theoretical and empirical generalizability.

## 6 Conclusion

The primary objective of this study was to examine the effects of AI-enabled distance learning platforms on adolescents’ academic performance and mental health in resource-scarce contexts, with a particular focus on educational equity and psychological wellbeing. The empirical findings indicate that participation in AI-supported learning was associated with substantial improvements in academic performance, alongside consistent reductions in anxiety and depressive symptoms. Although changes in emotional regulation did not reach statistical significance in the short term, the overall pattern of results suggests that AI-enabled platforms may exert differentiated effects across academic and psychological domains. Specifically, the findings highlight the capacity of AI-supported learning environments to alleviate affective distress linked to academic pressure while simultaneously enhancing learning outcomes. Through mechanisms such as personalized learning pathways, real-time feedback, and structured learning support, AI platforms appear to reshape both cognitive engagement and emotional experience, extending beyond traditional instructional approaches. These results contribute to ongoing theoretical discussions in educational technology by demonstrating that AI-based learning interventions may function not only as instructional tools but also as contextual supports for students’ psychological wellbeing in disadvantaged settings.

In relation to existing research, this study advances the literature by empirically examining the intersection of AI-enabled education and student mental health within a single analytical framework. Prior studies have often addressed academic performance or psychological outcomes in isolation, whereas the present findings suggest that AI-supported distance learning may simultaneously influence both domains. The absence of a short-term effect on emotional regulation does not undermine the potential psychological value of AI interventions; rather, it underscores the complexity and relative stability of regulatory capacities compared to affective symptoms such as anxiety and depression. This distinction underscores the need for longer term and more developmentally sensitive research designs to assess whether sustained exposure to AI-supported learning environments can foster self-regulatory skills over time. Future studies would also benefit from incorporating more comprehensive and multidimensional mental health assessment tools to capture nuanced psychological change. Moreover, extending the duration of intervention and follow-up periods would allow for a more robust evaluation of long-term academic and mental health trajectories. Overall, this study provides context-sensitive empirical evidence that AI-enabled distance learning can support academic achievement and reduce psychological distress in resource-scarce environments, while also highlighting the importance of integrating technological innovation with sustained psychological support to promote educational equity and mental health sustainability.

## Data Availability

The raw data supporting the conclusions of this article will be made available by the authors, without undue reservation.
